# Improved Learning and Memory in Aged Mice Deficient in Amyloid β-Degrading Neutral Endopeptidase

**DOI:** 10.1371/journal.pone.0004590

**Published:** 2009-02-25

**Authors:** Thomas Walther, Doris Albrecht, Matthias Becker, Manja Schubert, Elena Kouznetsova, Burkard Wiesner, Björn Maul, Reinhard Schliebs, Gisela Grecksch, Jens Furkert, Anja Sterner-Kock, Heinz-Peter Schultheiss, Axel Becker, Wolf-Eberhard Siems

**Affiliations:** 1 Department of Cardiology, Charité - University Medicine Berlin, Campus Benjamin Franklin (CBF), Berlin, Germany; 2 Centre for Biomedical Research, Hull York Medical School, Hull, United Kingdom; 3 Institute of Neurophysiology, Charité, Campus Mitte (CCM), Berlin, Germany; 4 Leibniz-Institut für Molekulare Pharmakologie (FMP), Berlin, Germany; 5 Paul Flechsig Institute for Brain Research, University of Leipzig, Leipzig, Germany; 6 Institute of Pharmacology and Toxicology, Otto-von-Guericke-University of Magdeburg, Magdeburg, Germany; 7 Freie Universität Berlin, Berlin, Germany; Mental Health Research Institute of Victoria, Australia

## Abstract

**Background:**

Neutral endopeptidase, also known as neprilysin and abbreviated NEP, is considered to be one of the key enzymes in initial human amyloid-β (Aβ) degradation. The aim of our study was to explore the impact of NEP deficiency on the initial development of dementia-like symptoms in mice.

**Methodology/Principal Findings:**

We found that while endogenous Aβ concentrations were elevated in the brains of NEP-knockout mice at all investigated age groups, immunohistochemical analysis using monoclonal antibodies did not detect any Aβ deposits even in old NEP knockout mice. Surprisingly, tests of learning and memory revealed that the ability to learn was not reduced in old NEP-deficient mice but instead had significantly improved, and sustained learning and memory in the aged mice was congruent with improved long-term potentiation (LTP) in brain slices of the hippocampus and lateral amygdala. Our data suggests a beneficial effect of pharmacological inhibition of cerebral NEP on learning and memory in mice due to the accumulation of peptides other than Aβ degradable by NEP. By conducting degradation studies and peptide measurements in the brain of both genotypes, we identified two neuropeptide candidates, glucagon-like peptide 1 and galanin, as first potential candidates to be involved in the improved learning in aged NEP-deficient mice.

**Conclusions/Significance:**

Thus, the existence of peptides targeted by NEP that improve learning and memory in older individuals may represent a promising avenue for the treatment of neurodegenerative diseases.

## Introduction

Neutral endopeptidase (NEP) E.C. 3.4.24.11, also known as neprilysin or enkephalinase A and abbreviated NEP, is widely accepted as one of the most prominent known enzymes for initial amyloid-β peptide (Aβ) degradation [1]–[5]. This has been basically described *in vitro* by Howell *et al.* [6] and *in vivo* by Iwata *et al.* [7], who reported human Aβ (hAβ) degradation through limited peptidolysis conducted by NEP. It has been further demonstrated that pharmacological interventions can be used to modify the Aβ concentration via NEP activity. For example, NEP inhibitor infusions in the rat brain paralleled by hAβ gavages resulted in pathological deposition of Aβ [8]. This is all the more important, since cerebral NEP is modulated by several factors such as age, behavior, and environment and can thus significantly modify Aβ concentrations [8], [9]. NEP was found to catabolize not only monomeric, but also oligomeric forms of Aβ [10].

The capacity of NEP to proteolyze Aβ was further confirmed by findings that NEP-deficient mice demonstrated higher cortical Aβ levels than wild-type ones, and human Aβ was more slowly degraded when injected into NEP-deficient mouse brains than into corresponding wild-types [11]. However, a very recent publication showed that the learning abilities of young NEP knockout mice were unaltered, in spite of elevated Aβ levels in the brain of these mice [12].

Surprisingly, although Alzheimer's disease is common in old age, none of the previous research investigated whether the increased Aβ levels observed in the NEP-deficient mouse brain were accompanied by a significant impairment in learning and memory in older animals.

Thus, using old NEP-deficient mice of different ages and behavioral, immunohistological, and electrophysiological approaches, it was the aim of our study to clarify the impact of NEP deficiency for the initial development of dementia-like symptoms in mice.

## Results and Discussion

**Figure 1:**
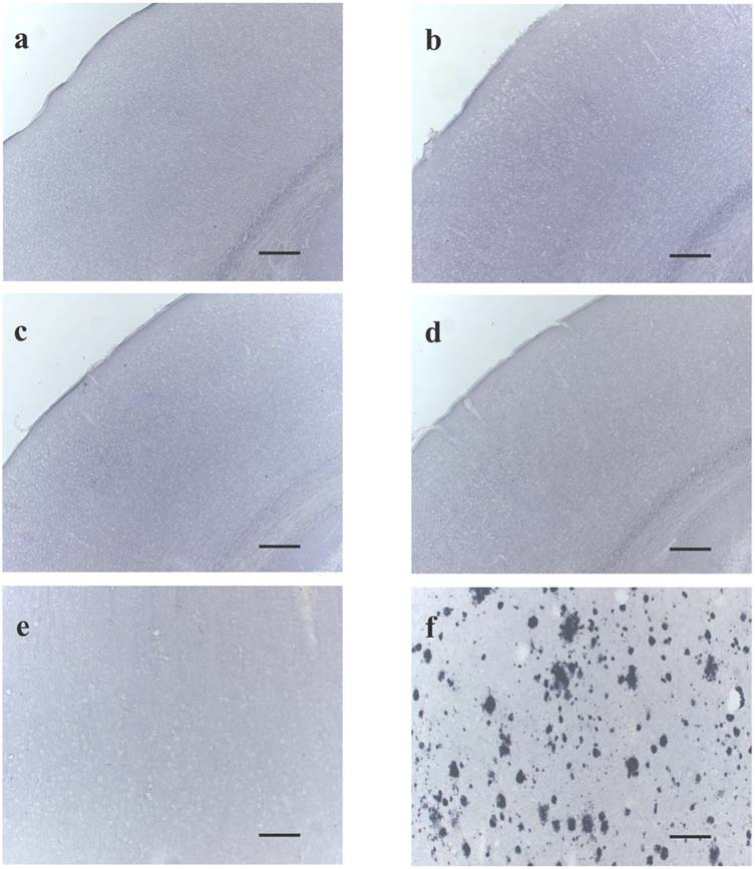
Cortical Aβ deposition in brains of humans and mice. Representative examples of immunostaining for Aβ in brain sections obtained from 6-month-old (b) and 24-month-old NEP-deficient mice (d) and corresponding age-matched wildtype controls (a,c), and from post mortem human brain material of a 75-year-old Alzheimer patient (f) and a 31-year-old human control (e). Images shown in a–d represent the mouse somatosensory cortex (barrel field), while human brain sections (e,f) were obtained from the temporal cortex. All sections were immunostained under the same conditions and in the same experimental session with the biotinylated primary antiserum 4G8 which is known to react with both human and murine Aβ peptides. Scale bar represents 200 µm.

Our first experiment focused on detecting possible Aβ depositions in NEP-deficient brains of mice at very different ages. Basically, these knockout mice were characterized by lower blood pressure and an enhanced lethality to endotoxin shock [13], [14]. Using a monoclonal antibody against human and murine Aβ [15], we failed, even in very old NEP-deficient mice, to stain Aβ deposits (**Figure 1b and d**), which is a classical morphopathological feature detectable in brains of Alzheimer patients (**Figure 1f**). As expected, neither the cortex of wild-type mice (**Figure 1a, c**) nor that of a person killed in an accident stained positively for Aβ deposits (**Figure 1e**).

**Figure 2:**
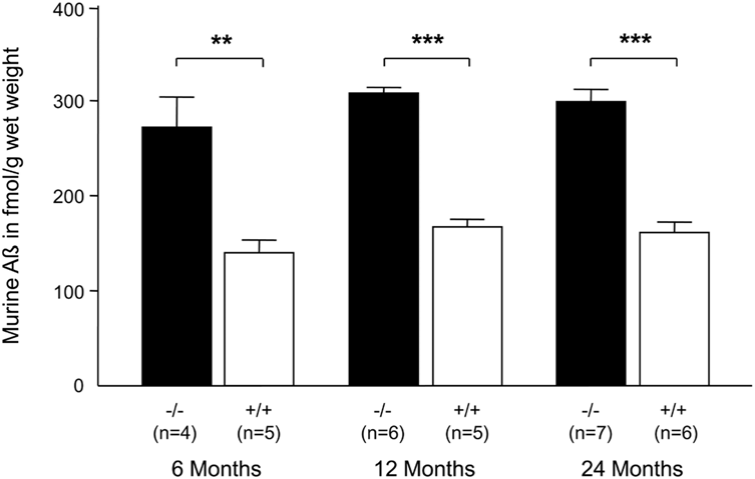
Aβ accumulation in brains of NEP-deficient mice. ELISA-measured murine Aβ(1–40) accumulation in the cortex of 6-, 12-, and 24-month old mice (−/− = NEP knockout [black bars]; +/+ = wild-type [white bars]); n≥6; average±s.e.m.; all differences were statistically calculated by a Student's t test. **P<0.01, ***P<0.001.

To quantify the postulated endogenous Aβ accumulation in brains of these mice at different time points, including very old mice (two-year-old mice), we used an ELISA based on a description by Tucker *et al.* [16]. While elevated Aβ levels in very young NEP knockout mice were already described [11], [12], we, for the first time, observed significantly higher Aβ levels in NEP-deficient mouse brains at all investigated time points in comparison to their age-matched wild-type mice (**Figure 2**). This elevation in endogenous Aβ, but the absence of any deposition (**Figure 1b and d**), underlines the different aggregation tendency of murine Aβ compared to that in humans.

Our results seem to be in contrast to findings by Iwata *et al.* [7] and Dolev & Michaelson [17], who described plaque formation and fibrillization, respectively, of endogenous Aβ in rats and mice after treatment with thiorphan, a non-peptidic inhibitor of NEP. Consistent with previous studies showing a broad spectrum of peptidases inhibited by that peptidase inhibitor [18], we hypothesized that there were other factors inhibited by thiorphan as a requisite for murine Aβ accumulation.

**Figure 3:**
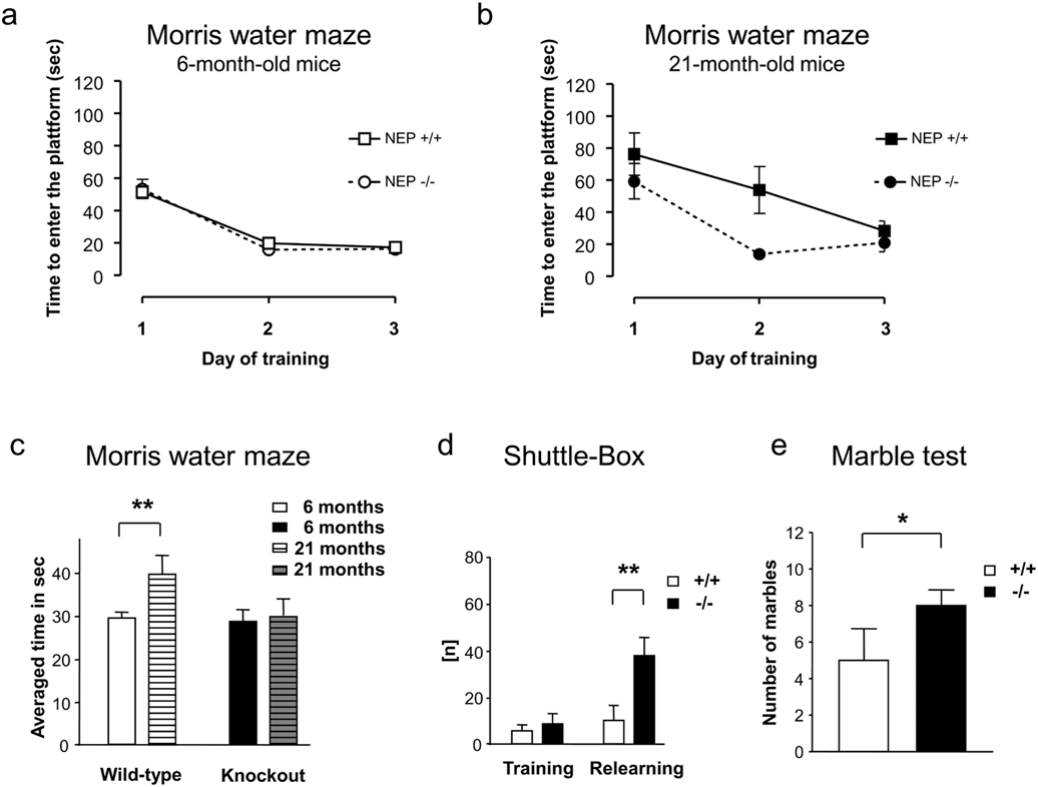
Behavioral studies in NEP-deficient mice and their wild-type controls. (a–c) Morris water maze: (a) Comparison of 6-month-old NEP-knockout mice (dotted line with open circles; n = 24) and their wild-type age-matched littermates (line with open quadrates; n = 19). Group means of escape latencies for the first trial per day to reach the platform ±s.e.m. in sec. Where standard variation is not visible it is within the symbols (b) Comparison of 21-month-old NEP-knockout mice (dotted line with filled circles; n = 12) and their wild-type age-matched littermates (line with filled quadrates; n = 9). Group means of escape latencies for the first trial per day to reach the platform ±s.e.m. in sec; statistical analyses by repeated measures of ANOVA (*P* = 0.016). (c) Comparison of time (in sec) animals needed to find the platform at both investigated time points; data were pooled for the three days of the experiment and plotted; statistical analysis by Student's t test (**P<0.01). (d) Shuttle box: group means of the number of conditioned reactions ±s.e.m. of 21-month-old NEP-knockout mice (filled bars; n = 12) and their wild-type littermates (open bars; n = 10) during a training interval on five training days in this two-way active avoidance paradigm. Statistical analysis by repeated measures of ANOVA (**P<0.01). (e) Marble burying test: Comparison of the number of marbles that have been buried by 21-month-old NEP-knockout mice (filled bars; n = 9) and their wild-type littermates (open bars; n = 11). Average±s.e.m., statistical analysis by Student's t test (*P<0.05).

Since already the accumulation of soluble forms of Aβ (monomeric and oligomeric) without plaque or fibril formation is assumed to induce neuronal pathology and consequently learning and memory deficits [12], [19], [20], we tested learning and memory performance in NEP knockout mice in comparison to wild-type animals. Six- and 21-month-old mice were subjected to behavioral tests, including spatial memory. Spatial memory is assessed by measuring the animal's ability to recognize objects or its sense of orientation in mazes. Whereas the genotypes did not differ at the age of six months (**Figure 3a**; Repeated measure of ANOVA with factors time, number of training sessions and genetic status: F1.41 = 0.072; *P* = 0.79; data shows the time the animals needed within the first trial per day to reach the platform), 21-month-old NEP-knockout mice required significantly less time than their wild-type littermates to find the hidden platform in a Morris water maze (**Figure 3b**; Repeated measure of ANOVA with factors time, number of training sessions and genetic status: F_1.19_ = 7.02; *P* = 0.016; data shows the time the animals needed within the first trial per day to reach the platform). Importantly, as expected, the 21-month-old wild-type mice required more time than the younger controls to find the hidden platform in a Morris water maze. However, this significant age-dependent impairment in spatial learning was not detected in NEP-deficient mice (**Figure 3c**), which suggests that a lack of NEP might sustain the learning ability (spatial memory) and the ability to efficiently complete the maze at an advanced age.

One aim was to determine whether these observed differences in spatial memory would be also evident in learning tasks with different motivation. In a two-way active avoidance task (shuttle box), mice had to learn to associate a sound (conditioned stimulus) delivered through rods on the floor of the shuttle box. They could avoid foot shock by moving to the opposite site of the chamber as soon as the conditioned stimulus was presented. The 6-month-old mice did not differ in any measured parameter (**data not shown**). We also found no significant differences in the training phase between the two groups of 21-month-old mice, but relearning performance was much better in NEP-deficient mice (**Figure 3d**). Thus, the age-dependent differences between the two genotypes further confirm the findings of the Morris water maze, and therefore two independent behavioral tests support the preserved learning in older mice deficient for the NEP gene. To test the emotional status of the knockout mice, both groups underwent the marble burying test. The data obtained suggests that knockout mice showed more anxious behavior towards novel objects than the wild-type strain (**Figure 3e**). Since it is commonly accepted that anxiety negatively affects learning performance [21], [22], improved learning behavior despite increased anxiety underscores the better performance of our NEP-deficient mice and indicates that elements of the mice's emotional behavior fail to dominate the differences in learning and memory in our experiments.

Cognitive processes such as learning and memory are believed to depend on changes in synaptic efficacy in certain key brain regions, including the hippocampus [23] and the amygdala [24]. To assess the importance of hippocampus- and amygdala-dependent forms of learning and synaptic plasticity in NEP knockout mice, we also examined the ability of hippocampal and amygdaloid synapses to support both paired pulse facilitation, a form of short-term plasticity, and long-term potentiation (LTP). Electrophysiological experiments were performed in anatomically well-characterized horizontal slices [25] as described in detail elsewhere [26]. Although several studies on aging and hippocampal LTP failed to demonstrate any age-related deficits using high-frequency stimulation (HFS) [27], theta burst stimulation (TBS) revealed age-related deficits in the induction of LTP [28]. The type of CA1-LTP induced by HFS seems to be genuinely different from LTP induced by learning processes or theta-patterned stimulation. Moreover, TBS-induced LTP in the CA1 region of the hippocampus depends only on N-methyl-D-aspartate receptors (NMDAR) [29] and NMDA receptor-dependent LTP appears to decline in the CA1 area of aged rats [30].

For this reason, we decided to induce TBS-LTP in the CA1 region of the hippocampus. After completing behavioral testing and a 3-week resting period, the animals were sacrificed and their brains subjected to LTP analysis.

**Figure 4:**
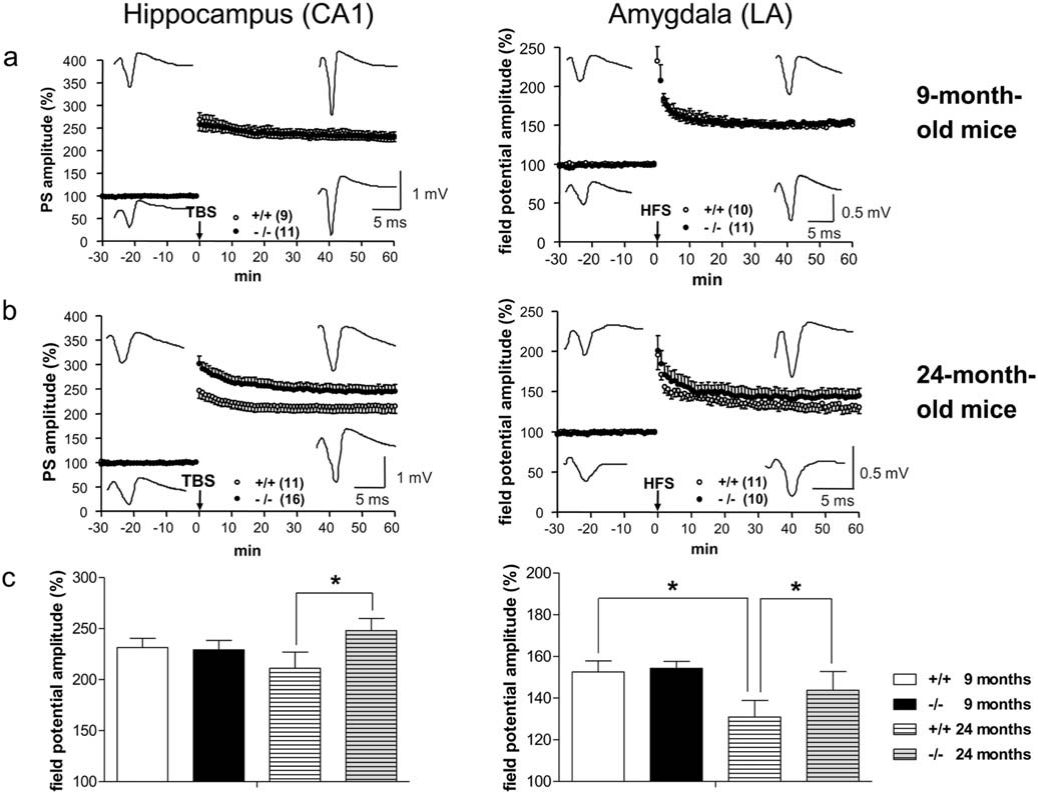
LTP studies in NEP-deficient mice and their wild-type controls. LTP in the hippocampus (CA1 region) and the amygdala (lateral nucleus of the amygdala (LA)) in adult (n = 7 mice) and aged NEP knockout mice (−/−; n = 7 mice) in comparison to wild-type (+/+) mice (n = 5 mice in each group). (a) 9-month-old mice: LTP magnitude (CA1: ○ +/+, n = 9 slices; • −/−, n = 11 slices; LA: ○ +/+, n = 10 slices, • −/−, n = 11 slices). Inserted representative records of PS potentials in the CA1 region before and after TBS and in the LA before and after HFS, above: obtained from −/−, below: obtained from +/+. (b) 24-month-old mice: LTP in both the CA1 region (○ +/+, n = 11 slices; • −/−, n = 16 slices) and the LA (○ +/+, n = 11 slices; • −/−, n = 10 slices). Data points in a and b represent mean amplitudes (mean±s.e.m.) normalized with respect to baseline values. Inserted representative records of PS potentials in the CA1 region before and after TBS and in the LA before and after HFS, above: obtained from −/−, below: obtained from +/+. (c) Bar histogram of data points shown in Figures 4a–b, averaged 56 to 60 min after TBS/HFS and normalized with respect to baseline (mean±s.e.m.). *P<0.05.

Except for impaired paired pulse facilitation in the CA1 region in the range of 10 to 70 ms in adult NEP knockout mice compared to the adult wild-type (*P*<0.05), we found no significant differences in basal transmission (input/output curves) or the strength of LTP between 9-month-old NEP-deficient and wild-type mice (CA1: TBS-induced LTP; LA: HFS-induced LTP; **Figure 4a**). In contrast, the 24-month-old knockout mice showed enhanced LTP in both the hippocampus and amygdala compared to their wild-type group (**Figure 4b**). Importantly, this enhancement of LTP in the CA1 region obtained in knockout mice compensated the age-related impairment of LTP obtained in the 24-month-old wild-type group compared to the 9-month-old controls (**Figure 4c, left diagram**). We also demonstrated for the first time an age-dependent decrease in LA-LTP that has been much less pronounced in preparations from brains lacking NEP (**Figure 4c, right diagram**). Paired pulse facilitation was significantly reduced in both structures of aged NEP-deficient mice, suggesting the involvement of presynaptic mechanisms (**data not shown**).

While murine Aβ accumulates but does not aggregate to macrostructures, the effect of oligomeric Aβ on neuronal function is thought to be pathological, because even very small aggregates affect neuronal function, learning and memory abilities in humans [19], [20]. Our behavioral animal studies may not support this conclusion, since NEP-deficient mice characterized by more Aβ, more oligomeric Aβ [12], but no macrostructural deposits do not demonstrate impaired learning in our 9-month-old mice. This finding is also supported by a recent publication showing unaltered learning abilities in very young (3–4 months) NEP-deficient mice [12], while it was significantly impaired if human Aβ (containing the Swedish double mutation) was expressed in addition in these knockout mice [13], [31].

Surprisingly, our data show that two-year-old knockout mice have an even greater ability to learn than their age-matched controls as demonstrated in two independent behavioral tasks (Morris water maze and shuttle box experiments). It is even more remarkable that the age-dependent decline in learning ability as shown for our wild-type animals is eliminated in aged NEP knockouts. These findings are strengthened by our electrophysiological findings. Since considerable effort has gone into attempts to relate hippocampal LTP to learning and memory [32], [33], we examined behavior and electrophysiology in the same subjects as an approach to link LTP with learning. Our data shows that sustained learning and memory in aged mice was paralleled by an improved LTP in horizontal brain slices of the hippocampal CA1 region and the lateral nucleus of the amygdala. The hippocampus is known to be involved in a variety of learning tasks, especially spatial learning in rodents. Long-term synaptic plasticity of glutamate-mediated transmission in the CA1 region of the hippocampus is believed to be an important process in learning and memory in vertebrates. In addition to the crucial role played by amino acid-mediated synaptic transmission, the hippocampus also receives a variety of non-amino acid-mediated synaptic input with the capacity to either promote or restrict the induction of long-term synaptic plasticity.

The underlying mechanisms leading to better learning and improved LTP are still speculative. However, this improved learning seems more likely to be an Aβ-independent effect in our mouse model. With respect to that conclusion, it is interesting to note that local Aβ accumulation following short-term inhibition of NEP by thiorphan infusion in either hippocampus [34] or the cortical ventricle [35] led to impaired learning and memory. These authors pharmacologically inhibited NEP in young rats for 4 weeks. Thus, their elegant approach to demonstrate that increased Aβ amounts are linked to impaired learning does not reflect on the situation in the aged NEP-deficient brain where, regardless of accumulating Aβ, the concentration of other peptides is altered. Moreover, comparing the results of thiorphan-treatment [11], [34], [35] with experiments in NEP-knockout mice, we have to consider that thiorphan inhibits further NEP-like peptidases whose activities are not impaired in NEP-deficient mice [18]. Nevertheless, further experiments should also prove a second possible explanation for the difference between genetically generated NEP deficiency and pharmacologically reduced NEP activity by thiorphan, namely that the unconditioned knockout of NEP results in several compensatory changes in the activities of peptidases discussed to be involved in Aβ degradation.

**Figure 5:**
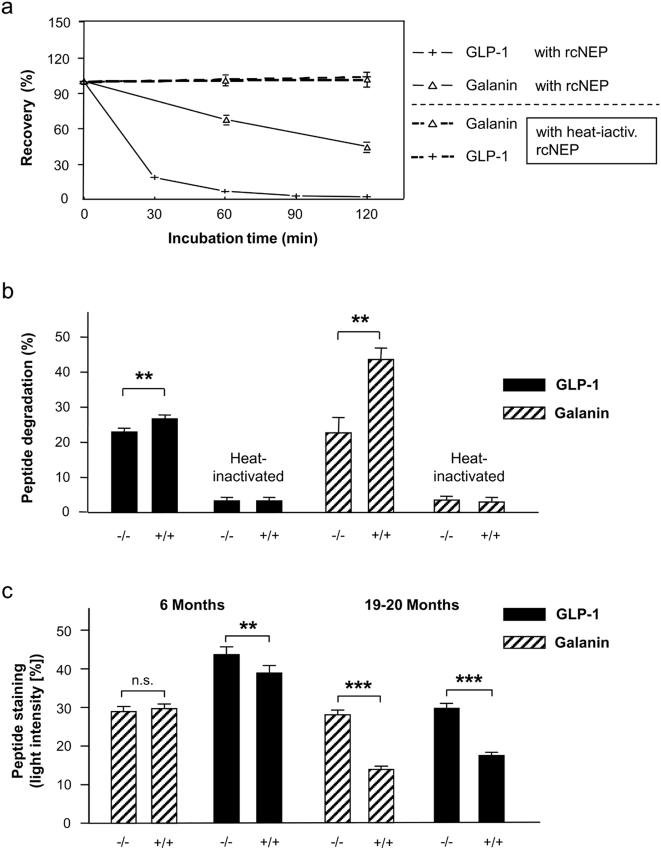
NEP-dependent neuropeptide degradation. (a) HPLC-monitored degradation (recovery in %) of GLP-1 and galanin (each 5 µM) over 120 min using recombinant (rc) NEP (20 ng; R&D Systems, Wiesbaden, Germany); mean values with s.e.m.; n≥3. The reactions were stopped by adding 0.35 M perchloric acid. In parallel assays, heat-inactivated probes (5 min at 90°C) were used as a control. After centrifugation of sedimented proteins, HPLC analyses were performed by isocratic elution as described by Siems *et al.* [45]. (b) HPLC-monitored peptide degradation (in %) of GLP-1 and galanin (5 µM) in brain membranes (0.5 mg protein/ml) of wild-type (+/+) and knockout mice (−/−) over 30 min. The reactions were stopped by addition of 0.35 M perchloric acid. In parallel assays, heat-inactivated NEP (5 min at 90°C) was used as a control. HPLC analysis was performed by isocratic elution as described [45]. The significance of differences was calculated by two-sided t test; n≥4; **P<0.01. (c) For galanin and GLP-1 immunohistochemistry, slices were incubated with the specific antibodies, followed by incubation with biotinylated anti-rabbit IgG. Immunoreactive products were visualized by the nickel ammonium sulfate-intensified diaminobenzidine reaction. Unequivocally stained regions in the murine cortex were localized and defined as ROIs (regions of interest), and luminometrically compared with adjacent regions without detectable staining (reference ROIs) (for both regions n = 40). The calculated differences were shown as mean±s.e.m.). Significant differences were calculated by Student's t test, indicated by **P<0.01, ***P<0.001.

It has been shown that NEP targets a variety of peptides involved in learning and memory such as oxytocin [36], NPY [37] or CCK [38]. Thus, marked improvement in learning and memory in very old NEP-deficient mice if compared with their aged-matched controls appears to be explained best by these NEP-degradable neuropeptides. Nevertheless, the known catalytic properties of NEP - it preferentially hydrolyses oligopeptides by cleaving on the N-terminal side of hydrophobic amino acid residues - and the knowledge of further potential learning-associated peptides [39]–[42] motivated us to test several other neuropeptides for being potential NEP substrates. Consequently, we initiated our own studies on the postulated NEP-dependent metabolism of GLP-1 and galanin. First results underscore the likelihood of our assumption. We observed *in vitro* a significant GLP-1 degradation by rcNEP (**Figure 5a**) and a reduced rate of GLP-1 degradation in brains lacking NEP (**Figure 5b**) comparable to that in wild-type membranes pretreated with NEP inhibitor candoxatrilat (**data not shown**). This is in agreement with a study of Hupe-Sodmann *et al.* (1995) postulating NEP peptidolytic activity for GLP-1 [39]. Our finding is all the more important, since GLP-1 has been found to improve associative and spatial learning if injected intracerebroventricularly [40]. Learning-associated properties are also known for galanin [41], [42], and due to its chemical structure, galanin is a prominent candidate for degradation by NEP. As shown in **Figures 5a and b**, we also identified galanin as a NEP substrate. Since, as we showed, GLP-1 and galanin are substrates for NEP, NEP deficiency may lead to an elevation of both peptides, thus improving learning and memory. To also test this hypothesis, we measured the concentration of both peptides in the cortex of NEP-deficient mice and their wild-type controls, and checked for possible age-dependency. While differences did not occur for galanin or have been minor for GLP-1 in 6-month-old mice that could not be discriminated in their learning capacity, significantly higher levels of both peptides were measured in aged NEP knockouts that are characterized by sustained learning capacity (**Figure 5c**). These findings further strengthen the hypothesis that both peptides could be involved in the improved learning in mice lacking enzymatic NEP activity.

Thus, known NEP-dependent peptides such as NPY, CCK, and oxytocin, and the two peptides GLP-1 and galanin, identified here as being NEP-dependent, appear to be potential candidates responsible for the improved learning and neuronal electrophysiology in NEP-deficient mice. Further studies will have to evaluate these candidates and may identify further peptides which accumulate under NEP deficiency and have beneficial effects on learning in aged mice.

In summary, our data provides the first direct evidence that the accumulation of endogenous Aβ does not *per se* cause Alzheimer-like symptoms in mice, contrary to findings with human Aβ expressed in transgenic mice (human APP-overexpressing mice) [43]. Importantly, this accumulation, without plaque formation, in contrast to the plaques observed in the human APP-overexpressing mice, did not hinder better learning abilities in aged NEP-deficient mice. Therefore, this data reveals the potential of peptides degradable by NEP to have a capability to improve cognitive properties in mammals and thus suggest that the pharmacological inhibition of NEP under normal conditions could sustain learning and memory in older individuals. However, NEP inhibition, although cardioprotective, could be a dangerous treatment strategy in humans, since the lack of central human NEP might lead to Aβ accumulation, plaque and fibril formation (in contrast to murine Aβ that does not aggregate to such macrostructures). On the other hand, the stimulation of central NEP may be beneficial in neurodegenerative disorders to reduce Aβ-derived depositions but may cause learning deficits by promoting the degradation of peptides with positive effects on LTP, learning and memory.

However, since our data strongly suggests the existence of these peptides targeted by NEP that improve learning and memory in older individuals, our study may open a promising avenue for the treatment of neurodegenerative diseases.

## Materials and Methods

### Animals

We used male NEP-knockout mice that were originally generated by Lu *et al.* [13] and maintained in the breeding stocks of T.W. at the Charité, Campus Benjamin Franklin (CBF), Berlin, Germany. The animals have been back-crossed to C57BL/6 background for more than 8 generations. Experimental animals were bred from parents, which were F2 after hemizygous mating. The lack of compensation for the loss of NEP by functionally and structurally related peptidases (APN, ACE) in the untreated animals shows that NEP-deficient mice and their corresponding wild-type strain constitute an excellent animal model to characterize NEP-related processes [44]–[47]. Animals were housed in litters at 22±1°C in a 12 h/12 h light/dark cycle with unrestricted access to food and water. Behavioral tests were performed between 9:00 a.m. and 12:00 p.m.

All experiments were performed according to the German National Guidelines for the Use of Experimental Animals.

### Human brain material

Post mortem human brain material was obtained from the Reference Center for Neurodegenerative Diseases Leipzig (courtesy gift from T. Arendt, University of Leipzig, Germany). Temporal cortices from a 75-year-old female Alzheimer patient (stage VI/C according to Braakk and Braak [48] and from a male 31-year-old patient (stage I-0-0) were used in this study.

### Tissue preparation and sampling of sections

Mice were deeply anesthetized and transcardially perfused with saline containing heparine, followed by fixative (4% paraformaldehyde in 0.1 M phosphate buffer, pH 7.4). Brains were removed from the skull and post-fixed in the same fixative overnight (for 18 hours) at room temperature. Following equilibration in 30% sucrose in phosphate buffer, 30-µm sections were cut in the coronal plane between bregma −1.06 mm and −2.30 mm according to the brain atlas of Franklin&Paxinos [49] and immersed in 0.1 M phosphate buffer (pH 7.4).

Post mortem human brain material used in this study was immersion-fixed in 4% paraformaldehyde for 4 days, followed by equilibration in 30% sucrose for 2 days, and stored in 30% sucrose in the presence of 0.1% sodium azide at 4°C. Thirty-µm sections were cut and immersed in 0.1 M phosphate buffer (pH 7.4), pending immunocytochemical analysis.

### Immunohistochemical procedures

#### Immunohistochemical staining of Aβ

Immunohistochemical staining of Aβ was performed on free-floating sections using the mouse monoclonal human Aβ protein antibody, clone 4G8, raised against amino acid residue 17–24 of the Aβ peptide (Signet, purchased from BioCat, Heidelberg, Germany).

Tissue sections were first treated with 85% formic acid for 10 min (mouse) and 20 min (human). Four immunoperoxidase labeling sections were thoroughly washed in Tris-buffered saline (TBS; pH 7.4) and pretreated with 5% normal goat serum in TBS also containing 0.3% Triton-X 100 (TBS-NGS-T) for 1 h. Sections were immunoreacted with the biotinylated primary antiserum 4G8 (1∶1000) for 20 h at room temperature. Thereafter, the sections were rinsed in TBS containing 2% bovine serum albumin (BSA) and then incubated for 1 h at room temperature with avidin–biotin peroxidase complex at room temperature for 1 h. Immunoreactivity was visualized by using 0.025% 3.3′-diamino-benzidine (DAB; Sigma, Deisenhofen, Germany); in the presence of 0.001% hydrogen peroxide diluted in 0.05 M Tis-HCl buffer (pH 8.0). For qualitative analysis, the reaction was intensified adding nickel–ammonium sulphate hexahydrate (0.05%; Merck, Darmstadt, Germany) to the DAB reagent. Finally, the sections were mounted onto gelatine-coated glass slides, air-dried, and covered with Enthelan.

Omission of the primary antibodies in control slides resulted in the absence of any immunoreactivity.

To minimize variations in experimental conditions, both human and murine brain sections of all specified ages were incubated with the same solution of antibodies and treated altogether in one experimental session. Stained brain sections were analyzed using a Zeiss Axioplan 2 light microscope including a Sony DXC-930P color video camera system (Zeiss, Jena, Germany).

#### Immunohistochemical staining of glucagon-like peptide 1 and galanin

For galanin and glucagon-like peptide 1 (GLP-1) immunohistochemistry, the ABC (avidin-biotin-horseradish peroxidase complex) method was performed. Sections were incubated with rabbit polyclonal anti-galanin antibodies (1∶100; Biozol, Eching, Germany ) or rabbit polyclonal anti-GLP1 antibodies (1∶100; Biozol, Eching, Germany) for 3 days at 4°C, followed by sequential incubation with biotinylated anti-rabbit IgG (Vector Labs., Burlingame, CA, USA) for 1 h and with ABC complex for 1 h at room temperature. Immunoreactive products were visualized by the nickel ammonium sulfate-intensified diaminobenzidine reaction. The sections were dehydrated in a graded series of alcohol, cleared in xylene, and cover-slipped with Entellan (Merck, Darmstadt, Germany).

### Amyloid β peptide measurement

Mice were killed by decapitations, the brains were removed rapidly, and the corresponding brain regions were prepared as described on chilled Petri dishes [50], weighed and immediately frozen on dry ice. For further treatment, tissues were rethawed in a 30-fold volume of ice-cold 1% (v/v) trifluoracetic acid, and homogenized at 4°C using a glass-teflon potter (Satorius-S, Göttingen, Germany). After centrifugation at 4°C and 17,000×g for 20 min, the supernatants were directly subjected to solid-phase extraction.

Sep-Pak cartridges (octadecasilyl-silica; C18 Sep-Pak Vac 3cc, Waters Corporation, Milford, USA) were rinsed first with 4 ml acetonitrile, followed by a 20 ml rinse with 1% (v/v) trifluoroacetic acid. After sample (see above) application, the cartridges were washed with 15 ml 1% (v/v) trifluoroacetic acid. Finally, the peptides were eluted with 4.3 ml acetonitrile: 1% (v/v) trifluoroacetic acid (3∶2 v/v) at flow rates of 0.7 ml/min. The eluate was dried overnight using a rotation vacuum concentrator (Alpha-RVC, Christ, Germany). The probes were stored until used at −20°C.

According to the manufacturer's manuals, murine Aβ(1–40) concentrations were exemplarily measured by a sandwich enzyme-linked immunosorbent assay (human/rat Aβ(1–40) ELISA kit; Wako Pure Chemical Industries, Osaka, Japan). The extraction recovery was 66.8% for murine Aβ(1–40) after spiking with the peptides. The dose-response curves paralleled the sample dilution curves. The cross-reactivity of murine Aβ(1–40) was 119% in this ELISA kit using anti-human Aβ(1–40) antibodies and human Aβ(1–40) as standards (the kit uses the following antibodies: anti human Aβ(1–40) MoAb clone no. BA27 Fab-HRP; and anti human Aβ(11–28) MoAb clone no. BNT77). Antecedent experiments with murine Aβ(1–40) (not shown) and confirmed the aptitude and linearity of the assays. All differences were statistically calculated by a Student's t test.

### Peptide degradation studies

The production of brain membranes and the HPLC-monitored peptide degradation procedures were performed as described in detail by Siems *et al.* [45]. The peptides (galanin, GLP-1) were purchased from Bachem (Weil am Rhein, Germany) or Phoenixpeptide (Karlsruhe, Germany). The recombinant NEP was obtained from R&D Systems GmbH (Wiesbaden, Germany). The specificity of NEP-caused degradation was confirmed by 10 µM of the NEP inhibitor candoxatrilat (Pfizer, Karlsruhe, Germany).

### Quantification of GLP-1 and galanin

Unequivocally stained regions for either GLP-1 or galanin in the murine cortex were localized and defined as ROIs (regions of interest). Furthermore, adjacent regions without detectable staining were defined as “reference ROIs” (both n = 40). Luminosities of the localized regions were measured using the software of the confocal microscope LSM510-META (Carl Zeiss MicroImaging GmbH, Jena, Germany) whereby the “reference ROIs” were set as 100%. The calculated differences reflect the staining of both neuropeptides. The antibody-evoked specificity of staining ensured the specificity of the method.

### Behavioral tests

#### Morris water maze

Sets of neurological and behavioral examinations demonstrated no differences in sensorimotoric or motivational functions between NEP knockout and wild-type mice. Thus, the hidden-platform test was performed in this study.

The Morris water maze was a round pool (1 m in diameter, 46 cm high) filled with water (20°C) to a depth of 26 cm. Different patterns were assigned to each segment for navigation. The platform (10 cm in diameter) was submerged by 0.2 cm. Water was made opaque by adding SAKRET© (SAKRET Trockenbaustoffe Europa GmbH, Wiesbaden, Germany) dispersion color. For three consecutive days, each mouse was placed in the pool six times at different starting points with a 20-min interval between trials at different places (training). Mice were given 120 sec to swim. After locating the platform, mice were allowed to remain on it for 15 sec before being returned to their home cages. Mice that did not locate the platform were placed on it and scored as 120 sec. Escape latencies for individual mice were averaged from each daily trial and for the time the animals needed within the first trial per day to reach the platform. Age-dependent differences were statistically calculated by a Student's t test. Genotype-dependent differences over time were calculated by a two-way ANOVA test. In an independent experimental setting different swimming speed between the two genotypes was excluded (NEP knockout: 13.1±1.5 vs. wild-type: 14.4±1.2 m/min).

#### Shuttle box experiments

The automatic shuttle box (0.35×0.13×0.15 m) was divided into two identical compartments separated by a 5 cm hurdle. The conditioned stimuli were light (40 W bulbs located on the central ceiling of each compartment) and a sound produced by a buzzer. The unconditioned stimulus was an electric stimulation of 0.2–0.4 mA depending on the individual sensitiveness of the animal (discernible reaction of the animal to the stimulation) whilst remaining below the vocalization threshold, delivered through stainless steel rods covering the floor (50 Hz, impulse widths 10 ms, pulsatile direct current). The conditioned stimuli - unconditioned stimulus interval was 4 sec. The stimuli were switched off when the mouse had moved to the goal compartment. One trial was limited to 20 sec if the animal failed to react within this period. The mean interval between trials was 30 sec. Prior to the first session, the mice were allowed to explore the box for 5 min. The training session involved 80 light/foot shock combinations. The next day, a relearning session was performed under identical conditions after one minute of habituation. The number of conditioned reactions (reaction time <4 sec) and intertribal activity were recorded. In-time escape from the shock-prone compartment was evaluated as a positive trial.

In a second shuttle-box experiment, another group of animals was tested on 5 consecutive days in the shuttle box. The animals were given 30 light/foot shock combinations a day. Test conditions (current, stimuli, stimuli intervals, and habituation) were identical to those described above. Significant differences were calculated by a Student's t test.

#### Marble burying test

The marble burying test is a validated model for object-related anxiety and compulsive-like behavior [51]–[53]. This test was performed in Macrolon II cages. The floor of each cage was covered with 5 cm of commercial bedding material (Rettenmaier GmbH, Rosenberg, Germany) on which 12 glass marbles (diameter 1.2 cm) were spaced. The mice were placed individually in a cage and left undisturbed for 30 min. The room was illuminated with standard fluorescent strip lights. After the test, the number of buried marbles (more than 75% of the marble volume) was counted and the bedding changed. The marbles were washed with 70% ethanol and dried prior to the first test and after each test. Significant differences were calculated by a Student's t test.

### Electrophysiology

Wild-type and knockout adult (9-month-old) or aged (24-month-old) mice were used in electrophysiological experiments. As described earlier in detail [54], the mice were anesthetized and decapitated. Their brains were rapidly removed and placed in ice-cold artificial cerebrospinal fluid (ACSF) with the following composition: NaCl: 124 mM; KCl: 3 mM; NaHCO_3_: 26 mM; Na_2_HPO_4_: 1.25 mM; MgSO_4_: 1.8 mM; CaCl_2_: 1.6 mM; glucose: 10 mM. The slices were placed in an interface chamber and allowed to equilibrate for 120 min at 34°C. They were superfused continuously with ACSF (1.5 ml/min). The pH was maintained at 7.4 by equilibration of the solution with 95% O_2_ and 5% CO_2_. Extracellular recordings from the lateral nucleus of the amygdala (LA) and CA1 region were made in parallel in different slices. Although we obtained similar results in *in vitro* experiments investigating long-term depression of activity in the LA recorded either intracellularly or extracellularly [26], it should be considered that the negative wave recorded extracellularly in the LA reflects a summation of both EPSPs and synchronized action potentials (population spike component; PS) [55], [56]. Watanabe *et al.* [55] have carried out intracellular recordings of evoked potentials and confirmed that the latency of peak negative field potentials (5–6 ms) corresponds well with that of intracellularly recorded action potentials, indicating that the extracellularly recorded sharp negativity seems to be a population spike. We therefore preferred to analyze the amplitude of field potentials in the present study. Additionally, the slope measure in the lateral amygdala is more sensitive to variability and noise in the signal, making it more difficult to analyze [55]. Thus, we decided to record PS also in the CA1 region of the hippocampus, to get a better comparison of results between these two limbic structures.

The input/output (I/O) response curves were constructed by varying the intensity of single-pulse stimulation (interstimulus interval: 10 sec; pulse duration: 100 µsec) and averaging 6 responses to each intensity. To evaluate short-term synaptic interactions, paired-pulse stimuli were delivered with interstimulus intervals ranging from 10 to 500 ms. To induce LTP in the CA1 region of the hippocampus, we stimulated Schaffer collaterals with a bipolar electrode placed on the surface of the slice to record population spikes (PS) in the pyramidal layer of the CA1 area. The stimulus was adjusted to elicit a PS at 30% of the maximal response, which was fixed at this level throughout the experiment. In different slices of the same mice, we recorded field potentials in the caudoventral part of the LA by stimulating fibers running through the external capsule. Since the LA does not have a clear architecture like the hippocampus and theta burst stimulation (TBS) was not able to induce stable LA-LTP in aged mice, we adjusted the stimulus to elicit a field potential at 50% of the maximal response and used high frequency stimulation (HFS) to induce LA-LTP. To express and compare changes of the field potentials (LA) or PS amplitude (CA1) between the animal groups, we averaged responses from the 56–60 min period after HFS and TBS. While HFS was induced by 2×100 Hz, duration 1 sec, 30 sec apart, TBS consisting of two sets of five theta bursts separated by 30 sec (2×5 TBS) was used with each burst involving four pulses at 100 Hz delivered at an interval of 200 ms. The significance of the changes was assessed by the Mann-Whitney *U* test using a commercial statistics package (Graph Pad prism 5.0).
